# Ultrasound-Guided Transverse Thoracic Muscle Plane Block (TTMPB) for Post-Sternotomy Pain: A Randomised Controlled Trial

**DOI:** 10.21315/mjms-01-2025-065

**Published:** 2025-12-31

**Authors:** Navkiran Singh Gill, Ariffin Marzuki Mokhtar, Huda Zainal Abidin, Chong Soon Eu, Yusrina Zahari, Mohamad Hasyizan Hassan

**Affiliations:** 1 Department of Anaesthesiology and Intensive Care, School of Medical Sciences, Universiti Sains Malaysia, Health Campus, Kelantan, Malaysia; 2 Department of Anaesthesiology and Intensive Care, National Heart Institute, Kuala Lumpur, Malaysia; 3 Department of Anaesthesiology and Intensive Care, Hospital Sultan Idris Shah, Serdang, Selangor, Malaysia

**Keywords:** pain, Transverse Thoracic Muscle Plane Block, Visual Analogue Scale, incentive spirometry, patient controlled analgesia, fentanyl

## Abstract

**Background:**

Poorly controlled acute surgical pain after cardiac surgery results from extensive tissue injuries and associated with high pain intensity and leading to chronic pain and excessive opioid use. The aim is to analyse the pain scores, patient controlled analgesia (PCA) fentanyl requirement and spirometry values in Transverse Thoracic Muscle Plane Block (TTMPB) vs. conventional opioid strategy in patients who underwent cardiac sternotomy surgeries.

**Methods:**

This is a randomised controlled trial involving 40 adult patients who underwent elective cardiac surgery with sternotomy, receiving either TTMPB (B, *n* = 20) or control (A, *n* = 20). We measured mean pain scores at rest and on movement postextubation using the Visual Analogue Scale (VAS), mean PCA fentanyl consumption, and incentive spirometry volume at 0, 3, 6, 12, 18, 24, and 48 h postextubation. Analysis was done using the repeated measure ANOVA.

**Results:**

At rest, there was a significant reduction in pain score across different time points observed between both groups (F [6, 228] = 3.180, *P* < 0.05). On movement, a significant interaction between the TTMPB treatment and pain score at movement across different time points (F [6, 228] = 2.249, *P* < 0.001) was shown. There was also a significant reduction in PCA fentanyl consumption across different time points (F [6, 228] = 2.080, *P* < 0.05). Similar outcomes were also observed in spirometry volume changes across time points between the two study groups (F [6, 228] = 10.855, *P* < 0.001).

**Conclusion:**

The administration of the TTMPB resulted in significantly reduced pain scores at rest and movement, reduced the mean PCA fentanyl consumption and showed better incentive spirometry volume postextubation compared to the control group. This study supports TTMPB as efficient postoperative analgesia for post-cardiac surgery.

## Introduction

Cardiac surgeries are becoming increasingly prevalent all over the world. Patients undergoing cardiac surgeries are at particularly high risk of perioperative complications both from general anaesthesia and the surgical procedure itself, with severe pain arising from somatic and visceral tissues. Routine perioperative pain management related to cardiac sternotomies commonly utilise strong acting opioids as part of pain management strategies despite commonly known adverse effects like respiratory depression, postoperative nausea and vomiting. Poorly controlled acute surgical pain can be highly debilitating and has been associated with chronic pain.

In recent years, much attention has been given to ultrasound-guided regional nerve blocks targeting fascial planes. The Transverse Thoracic Muscle Plane Block (TTMPB) utilises local anaesthesia agents deposited in the plane between the internal intercostal muscle and the transverse thoracic muscle. The TTMPB is administered at 1 cm lateral to the sternum at level T4 intercostal space. This analgesic block is performed under ultrasonographic guidance, using aseptic technique, and can be performed via a longitudinal or transverse approach. The TTMPB aims to provide analgesia to the anterior chest wall, specifically to reduce pain from sternotomy/sternal retraction and even metal wires holding the anterior chest wall after surgery.

As previous studies comparing TTMPB vs. opioids, had shorter duration of study as patient post operative assessment was only up to 24 h and also lacked objective judgement of pain scores as pain was assessed intraoperatively by sympathetic response to surgical incision which could be contributed by multiple other factors such as hydration and volume status as well as sympathetic response from other non-incision related surgical stress (1). We recorded baseline preoperative pain scores, spirometry volumes and compared with postoperative readings in addition to evaluating postoperative opioid requirements at 6 h intervals until 48 h. This randomised controlled trial aims to compare whether TTMPB vs. opioids were more effective at reducing pain scores at rest and movement, PCA fentanyl usage, as well as improved spirometry volumes.

## Methods

This was a single-centre, double-blinded, prospective randomised controlled trial, and the study was approved by the Institutional Ethical Board of Universiti Sains Malaysia (USM/JEPeM//22010018). Written informed consent was obtained from patients.

**Figure 1:**
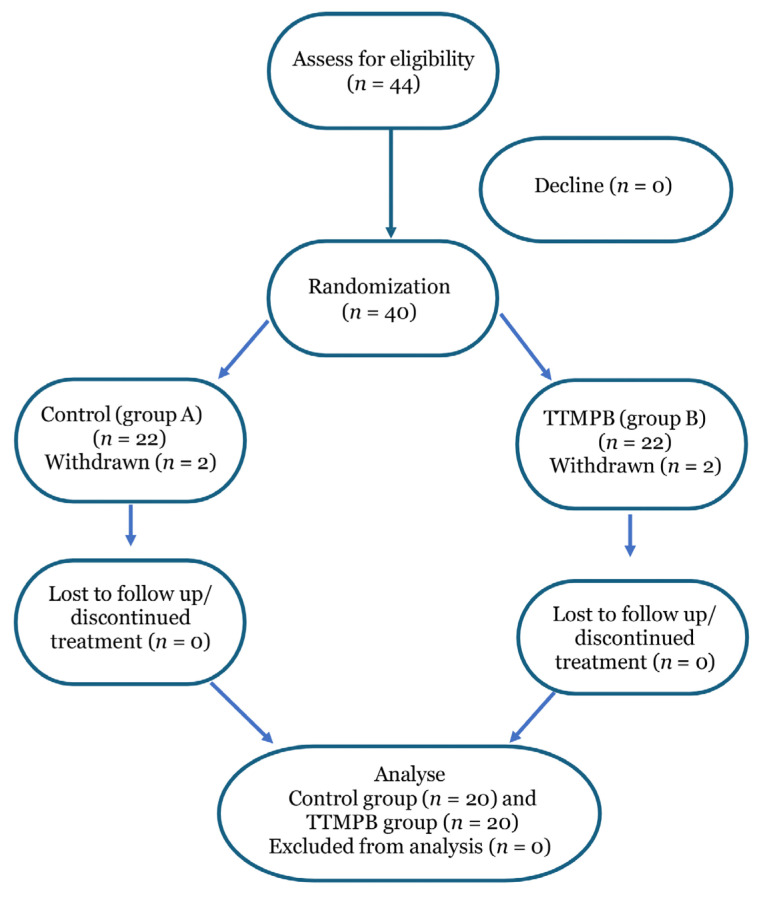
Consort flow diagram for the study

Adult patients who underwent elective cardiac surgeries were recruited (Figure 1). A total of 40 patients with American Society of Anesthesiologists II and III who underwent midline sternotomy were recruited. The exclusion criteria included allergy to study medications, high risk of bleeding (defined as INR > 1.5), long bypass time (> 150 min), left coronary main stem disease, emergency surgery, redo chest surgery, or ejection fraction 30% or below (echocardiography one day before surgery). The withdrawal criteria were: patient or caregiver refusal to participate in the study; a life-threatening event from the intervention; reopening the chest for surgery; non-adherence to the study protocol by staff or the patient; collection of patient identifiers; and any need to unblind the patient to receive appropriate treatment.

Block randomisation was applied to randomise the participants into two groups: either receive TTMPB (Group B) or control (Group A). The patients, the anaesthetist in charge of the operating theatre, the cardiothoracic surgeon, the investigators, and the outcome assessor were blinded to the study drugs. The blinded outcome assessor was a skilled cardiothoracic intensive care unit nurse with 10 years of experience, who measured the patient’s pain scores, fentanyl requirement, baseline and postoperative spirometry values. Baseline pain score, spirometry values, blood pressure, heart rate, oxygen saturation, and respiration rate were recorded during the preoperative visit.

On the day of intervention, the patient was called upon to operating theatre 1 h before operating time. The patient was placed on the operating table and standard American Society of Anesthesiologists (ASA) monitoring was applied. Adequate intravenous lines, arterial line, and central venous line were inserted via aseptic technique using ultrasonographic guidance under local anaesthesia. Pre-oxygenation was done to achieve an end-tidal oxygen partial pressure of 90 to 100 mmHg. The patient was then induced with intravenous fentanyl 5 to 10 mcg/kg, and titrated doses of intravenous propofol co-administered with intravenous midazolam. A bolus dose of intravenous rocuronium 0.9 to 1.2 mg/kg, followed by intubation with an appropriate cuffed size endotracheal tube. All participants received a dose of intravenous morphine at 0.1 mg/kg. Hemodynamic parameters were charted at 5 min intervals as per usual. General anaesthesia was maintained with a combination of medical air/oxygen and Sevoflurane, with a minimum alveolar concentration of 1.0. The patient also received standard active warming, temperature monitoring, and inotropes/vasopressors to ensure adequate mean arterial pressure (MAP) and perfusion to all organs. Midline sternotomy and planned surgical procedure was performed.

Group A, the control group, underwent surgery in accordance with “Standard Operating Procedures” and pre-existing guidelines. Postoperatively, after closure of skin incision by the surgeon, Group B patients received the TTMPB using levobupivacaine 0.25% at 20 mL bilaterally at 1 cm lateral to the edge of sternum at 4th intercostal space under aseptic technique and using ultrasonography guidance. Patient controlled analgesia (PCA) machine (Agilia^®^ SP PCA, Frenesius Karbi, Norge AS) using fentanyl at dilution of 20 mcg/mL was connected, which composed of background infusion of fentanyl 40 mcg/hr. The patient was extubated after fulfilling extubation criteria, as reviewed by the primary team and the consultant cardiac anaesthetist. Postextubation pain scores, PCA fentanyl bonus requirement and incentive spirometry (Voldyne 2500 Hudson RCI, Teleflex Medical, USA) volume were assessed at 0 h, 3 h, 6 h, 12 h, 18 h, 24 h and 48 h postextubation. The pain scores were charted using the Visual Analogue Scale (VAS), which is a standard pain measuring tool accredited by the International Association for the Study of Pain (IASP) (2).

The TTMPB procedure was performed by a single person, i.e., the researcher, who has more than 5 years of experience performing TTMPB, using aseptic technique under ultrasonographic guidance (Versana Active^™^ GE Healthcare, California, USA). A linear probe was utilised and placed in the transverse plane 1 cm lateral to edge of 4th intercostal space. With the internal intercostal muscle, transverse thoracis muscle as well as the internal thoracic artery and vein identified over the pleura, an 80 mm block needle was placed at the interfacial plane between the internal intercostal and transverse thoracic muscles using in-plane technique and localised confirmed using 3 mL of saline hydrodissection. Then, 20 mL of Levobupivacaine 0.25% was injected bilaterally according to the allocation. Apart from that, both groups received 10 mL of 0.25% levobupivacaine to cover pain on the side of chest and mediastinal drain by the surgeon.

### Sample Size and Statistical Analysis

We conducted a study to analyse postextubation pain scores, opioid requirement and incentive spirometry through regular time intervals from independent control and experimental subjects, with one control per experimental subject. Based on two mean formulas for repeated measures analysis of variance (ANOVA), between factors using G-power, with a size effect f of 0.35, power of study (80%), α error probability (0.05), number of groups (two), number of measurements (seven), and correlation among repeated measures (0.5), the highest number of patients required for the proposed objectives is 20 per group. With a 10% dropout rate, the sample size was 22 subjects per group, for a total of 44 subjects.

The analysis was performed using the IBM SPSS Statistics for Windows Version 21.0. Descriptive statistics were employed for selected variables. Categorical data were presented as frequencies and percentages and analysed using the Chi-square test. Numerical data were presented as means and standard deviations, and as medians and interquartile ranges whenever appropriate. Comparison of numerical data between two independent groups that are normally distributed was analysed using the independent *t*-test, and comparisons for skewed data were analysed using the Mann–Whitney test.

Post-mean extubation pain scores, PCA fentanyl requirement, and post-op spirometry volumes were analysed using two-way repeated measures ANOVA. All probability values are two-sided, and a level of significance of less than 0.05 (*P* < 0.05) were considered as statistically significant.

## Results

A total of 44 patients were included in the randomisation, with four dropouts due to re-sternotomy for bleeding and surgical site infection postoperatively; thus, there were 20 samples in each of the TTMPB and Control groups (Table 1). Overall, both groups were homogenous, as there was no significant difference in mean age, BMI, MAP, duration of surgery, mean cardiopulmonary bypass time, or baseline spirometry values between the two study groups (*P* > 0.05). Post hoc analysis with Bonferroni correction applied with no determined confounders. The incidence of postoperative pneumonia between the two groups was also not significant.

The first parameter assessed was the pain score after extubation at rest (Table 2) and on movement (Table 3). There was a significant reduction in pain scores at rest throughout the postextubation period, and subsequently lower pain scores in the first 24 h to 48 h of the intervention TTMPB group compared to the control group. A similar significant trend of improved pain control at movement, as shown in Table 3, is observed in the intervention TTMPB group compared with the control group. Satisfactory pain scores (pain of 3 or below) at rest and movement elicited in the treatment group as early as 3 h after extubation and persistently reduced to 48 h after extubation (*P* < 0.001), whereas the control group reached satisfactory pain scores much later throughout the study period.

The second parameter analysed is the PCA fentanyl requirement postoperatively, as seen in Table 4. While both groups were given the same background and opioid (fentanyl) on-demand bolus, the intervention group (TTMPB) showed lower total opioids (fentanyl) requirement compared to the control group at all time frames (*P* < 0.001). We also highlighted the fact that patients in the intervention group did not require PCA fentanyl at 48 h postextubation. There was no additional rescue analgesia op top of PCA fentanyl, such as tramadol or ketorolac, needed for both groups.

The final parameter analysed is the incentive spirometry. As shown in Table 5, incentive spirometry volumes postextubation were assessed for both the control and intervention groups at all time points. As observed, from 3 h postextubation onwards, there were significant differences between the study groups wherein, the postextubation incentive spirometry volumes were higher in the intervention (TTMPB) group right up to 48 h postextubation. In the control group, patients were unable to perform spirometry for the first 3 h after extubation due to drowsiness and pain upon movement. Spirometry values continuously improved over time points, signifying lower pain intensity, reduction of opioid use and improved general conditions after surgery in the interventional group.

We then moved on to analysing the relationship between the three study objectives and the intervention effect across groups, regardless of time (treatment interaction). According to the analysis in Table 6, at rest and during movement, significant reductions in pain scores were observed (*P*-values of 0.031 and 0.026, respectively) when mean (standard deviation [SD]) values were compared between the control and intervention groups. In similar trend, significantly reduced PCA fentanyl consumption was also observed when mean (SD) values between control and intervention with *P*-value of 0.006. However, when comparing spirometry values at treatment interaction, we noted a non-significant *P*-value of 0.051. But when we further explored changes in spirometry volume across different time points between the two study groups, we found a significant increase in spirometry values. (F [6, 228] = 10.855, *P* < 0.001). The treatment altered spirometry volumes at different time points.

There were no adverse reactions or complications, such as pneumothorax, haematoma, or local anaesthetic toxicity, encountered during the study. There were also no episodes of hypotension and bradycardia among those who received TTMPB.

## Discussion

This study was conducted to analyse the analgesic efficacy of TTMPB in adult patients undergoing sternal incisions, an important subset of enhanced cardiac recovery protocols worldwide. We are now evidently able to support the notion that TTMPB is indeed an effective technique in managing pain arising from cardiac sternotomy surgeries (1). This study demonstrated that TTMPB treatment improved pain scores at rest and during movement, reduced overall total PCA fentanyl usage, and increased incentive spirometry volumes achieved up to 48 h postextubation in adult patients undergoing elective cardiac sternotomy surgeries. It is also important to highlight the presence of postoperative infection (i.e., pneumonia), in the control group vs. the intervention group. This evidence is also supported by a recent meta-analysis of randomised clinical trials published in 2019 by Monahan et al. (3), that compared 13 randomised controlled trials consisting of 605 participants (312 to regional anaesthesia and 293 to comparator) and found administration of regional anaesthesia techniques (neuraxial blocks like spinal and epidural, peripheral nerve blocks, intravenous lignocaine infusion, intrapleural analgesia, wound infiltration) reduced pain scores up to 24 h after cardiac surgery. Comparatively, despite having the option of thoracic epidural for which inarguably provides excellent analgesia for cardiac sternotomy surgeries, the risk of epidural hematoma and epidural infections is higher considering heparinisation of patient intraoperatively and could prove detrimental to patients undergoing cardiac surgery thus also further supporting the role of regional anaesthesia techniques which are more superficial and done using ultrasonography enabling the anesthesiologist to have an objective assessment and able to provide targeted fascial plane blocks (4).

Effective pain control at rest and at movement is vital, ensuring patients remain calm with good orientation and cooperative as they recuperate from a major surgical stress (5). The chest wall has rich innervations of mechanoreceptors as well as nociceptors, divided into dermatomes originating respectively from their thoracic nerve roots, namely T1–T11. They are further divided according to the regions innervated, namely the anterior and medial as well as the lateral and posterior cutaneous intercostal nerves. The local anaesthetic administered in the fascial plane where these nerve fibres are located are able to inhibit initiation and transmission of nerve impulse at the level of the nociceptors (6). Several other studies have also proven the role of the TTMPB in reducing pain, namely Cakmak et al. (7) in Turkey, between January 2018 and March 2019 where a retrospective study was done to assess effectiveness of postoperative analgesia at by administration of levobupivacaine 0.25% at a dose of 0.5 mL/kg in 33 children undergoing cardiac sternotomy surgeries whereby, participants were divided into intervention and control groups (7). It was found that pain scores were significantly lower in the TTMPB group, intraoperatively and postoperatively, whereas the control group required higher doses of fentanyl. In addition, the time to extubation was much shorter in the TTMPB group (7). Similarly, Fujii et al. (8)conducted a pilot feasibility study in 2019 in London where a total of 19 patients were recruited whereby eight patients received the TTMPB treatment went on to displaying significantly lower pain scores at 12 h however not displaying much difference in postoperative hydromorphone administration with mean at 24 h was 1.9 mg (SD = 1.1) in the TTMPB group vs. 1.8 mg (SD = 1.1) in the control group (8). Although in our study, when analysed using repeated measure ANOVA, there was no interaction of pain scores at rest across groups with TTMPB intervention at different time points, but there was a significant reduction of pain scores at rest within the subjects, leading to our postulation of effective pain control.

As we set our sights to enhance the strength of our findings further, we charted and analysed the usage of PCA fentanyl postextubation for our patients. While intraoperatively, patient was loaded with intravenous morphine 0.1 mg/kg, fentanyl was chosen as our postoperative analgesia together with intravenous paracetamol 1 gram every 6 h and intravenous tramadol 50 mg every 8 h. As per our study protocol, PCA fentanyl was given as supplemental analgesia to maintain analgesic corridor as well as rescue analgesia in breakthrough pain for both the TTMPB and the control group. The pharmacology of fentanyl entails a strong opioid that is highly lipid soluble, potent, rapidly acting, shorter duration, with less fluctuations of blood pressure and heart rate, as well as fewer incidences of bowel ileus and postoperative nausea and vomiting compared to morphine (9–10). As evident above, the TTMPB group registered a significantly lower amount of fentanyl consumption postextubation compared to the control group up to 48 h of charting. It can also be observed that there was no PCA fentanyl consumption or usage at 48 h of charting in the TTMPB group, while the control group still required fentanyl at 48 h postextubation.

We then took a look at other articles and found similar findings. For example, Aydin et al. (1), in a total of 61 patients, showed a statistical difference in 24 h fentanyl consumption; median (IQR) requirements for the TTMPB were significantly lower compared to the control group (235 mcg vs 465 mcg) (1). Additionally, it was noted that the time to first rescue analgesic requirement was 19 ± 9 h and 7 ± 10 h in the TTMPB and the control group, respectively (1). Poor pain control post-cardiac sternotomy surgeries can limit tidal breathing, contribute to poor cough effort and impaired clearance of foreign debris, microorganisms, and mucus of the respiratory tract. This, in turn, could lead to atelectasis, hospital-acquired pneumonia that can cause a great degree of ventilation and perfusion mismatch as well as shunting with resultant hypoxemia and impaired minute ventilation leading to cardiorespiratory failure.

Breathing complications post coronary artery bypass graft come with a high healthcare cost (11). We therefore wanted to compare the effect of administration of the TTMPB vs. the conventional opioid strategy with regard to the ability of patients to perform incentive spirometry. As per our study findings, we note that the TTMPB group was able to perform better incentive spirometry postextubation at 3 h with a *P*-value of 0.01 (< 0.05) until 48 h postextubation. As seen using the repeated ANOVA measurement, the treatment significantly altered the incentive spirometry volumes across different time points between the intervention and control study group (F [6, 228] = 10.855, *P* < 0.001). We would like to add to the above postulations that none of the patients in the TTMPB group registered any incidence of postoperative pneumonia compared to the control group, with two incidences of pneumonia. As there were no significant or even high-quality methodological studies have been conducted to investigate the effects of cardiac sternotomy blocks on postextubation incentive spirometry, our study can be considered as the pioneer study to investigate this relationship. However, it will be pivotal to highlight the systematic review and meta-analysis done by Sullivan et al. (12) in 2021, comprising randomised controlled trials involving 3,776 adult patients that underwent cardiac, thoracic and upper abdominal surgeries, postulated that incentive spirometry alone did not significantly reduce 30-day postoperative pulmonary complications or 30-day mortality (12). An important point to focus on in this discussion is the safety in performing the TTMPB. The TTMPB targets anterior cutaneous nerves that are found in the plane between the internal intercostal muscle and the transverse thoracic muscle after they exit from the thoracic dermatome nerve roots. Although there are vital structures found in the proximity of the TTMPB plane vicinity, such as the internal mammary artery, vein, as well as the thoracic pleura, in our study, none of the control and the intervention group participants had any complications such as muscle hematoma, pneumothorax or even surgical site infection (1, 6). In a retrospective observational study recently published in 2022 done by Abdelbaser and Mageed (13), to assess the safety profile of ultrasound-guided TTMPB, conducted among 198 paediatric patients who received the TTMPB from a group of five anesthesiologists of varying degrees of experience, from highly experienced to less experienced anesthesiologists under supervision (13). It was reported that out of 198 patients, only one patient had a minimal unilateral subcutaneous hematoma that was self-limiting and resolved spontaneously, one patient had a puncture of pleura with no consequent pneumothorax or hemopericardium and one pericardial puncture that was managed conservatively (13). This was attributed to the loss of visualisation of the tip of the needle during advancement while the block was performed by the less experienced anesthesiologist. Other complications such as internal mammary artery injury, pneumothorax, hemopericardium or cardiac injury were not seen in this study. No episodes of hypoxia, hypotension, bradycardia, or local anaesthesia toxicity were reported, as the TTMPB is a fascial plane block, unlike a central neuraxial blockade, which causes significant pharmacological sympathectomy (13).

### Limitations

The limitation of our study was that it was conducted during the COVID-19 pandemic, therefore the patient’s spirometry volumes could have been affected if they had a history of COVID-19 pneumonia or an asymptomatic COVID-19 infection. Initially, we thought of including the paediatric cardiac population in our study, but due to COVID-19 restrictions, we decided to study adult patients, as they occupied the larger portion of the cohort of patients agreeing to elective surgery.

We also would like to highlight that the other limitation of this study is that it was a single-centre randomised controlled trial. More significant results would have been obtained if it had involved multi-centre studies, including cardiac-based centres that largely cater to this specific cohort of patients. Clinical trials with larger sample sizes will provide greater variation and further enhance the effects of this study.

Another very important point that was considered before the initiation of this study was whether TTMPB could be done pre-emptively before sternotomy, by the postulation that it would further aid reduction in overall intraoperative opioid usage. However, to the author’s knowledge and research, it will be difficult to assess pain scores directly during surgery, other than taking indirect measurements such as blood pressure and heart rate fluctuations as a surrogate for measuring sympathetic surge due to pain. These parameters are influenced by multiple factors during surgery, such as volume status, surgical manipulation of the thoracic cavity, and Trendelenburg positioning during sternotomy surgery.

## Conclusion

The administration of the TTMPB resulted in significantly reduced pain scores at rest and movement, mean PCA fentanyl consumption and showed better incentive spirometry volume postextubation compared to the control group. This study supports the use of TTMPB as an efficient postoperative pain management and potentially reduces the risk of postoperative pulmonary complications by improving postoperative lung volume early.

## Figures and Tables

**Table 1:** Participants baseline characteristics

Variables	Overall *n* (%)	Group		*P*-value

		Control *n* (%)	Intervention *n* (%)	
Age, year^a^	57.05 (12.86)	58.15 (9.60)	55.95 (15.65)	0.595^b^

Gender				
Male	25 (62.50)	14 (70.00)	11 (55.00)	0.327^c^
Female	15 (37.50)	6 (30.00)	9 (45.00)	

BMI, kg/m^2^ a	25.57 (4.15)	25.20 (3.42)	25.95 (4.83)	0.572^b^

MAP, mmHg^a^	89.60 (8.47)	91.00 (6.36)	88.20 (10.13)	0.302^b^

DOS, min^a^	198.80 (40.12)	196.75 (37.96)	200.85 (43.06)	0.751^b^

CPB time, min^a^	99.67 (16.50)	97.12 (18.91)	101.95 (14.14)	0.388^b^

ASA				
II	6 (15.00)	2 (10.00)	4 (20.00)	0.379^c^
III	34 (85.00)	18 (90.00)	16 (80.00)	

Pneumonia				
No	38 (95.00)	18 (90.00)	20 (100.00)	0.147^c^
Yes	2 (5.00)	2 (10.00)	0 (0.00)	

Spirometry, mL^a^	1,250.00 (277.35)	1,250.00 (334.43)	1,250.00 (214.60)	1.000^b^

a Mean (SD);

b Independent *t*-test;

c Chi-square significantat test for homogeneity;

* Statistically *P* < 0.05

BMI = body mass index; MAP = mean arterial pressure; CPB = cardiopulmonary bypass; DOS = duration of surgery; ASA = American Society Anesthesiologists; PCA = patient controlled analgesia

**Table 2:** Participants’ pain score postextubation at rest between the study groups

Pain score postextubation at rest	Overall^a^	Group		95% CI	*P*-value

		Control^a^	Intervention^a^		
0	5.08 (1.21)	5.65 (0.67)	4.50 (1.36)	4.73, 5.71	0.002*
3	3.63 (1.39)	4.55 (0.69)	2.70 (1.30)	3.29, 3.95	< 0.001*
6	3.33 (1.44)	4.30 (0.92)	2.35 (1.18)	2.99, 3.67	< 0.001*
12	2.85 (1.25)	3.75 (0.79)	1.95 (0.94)	2.57, 3.12	< 0.001*
18	2.45 (1.11)	2.95 (1.10)	1.95 (0.89)	2.13, 2.77	0.003*
24	2.35 (1.08)	2.95 (1.10)	1.75 (0.64)	2.06, 2.64	< 0.001*
48	1.85 (0.98)	2.35 (1.09)	1.35 (0.49)	1.58, 2.12	< 0.001*

a Mean (SD);

* Statistically significant at *P* < 0.05;

Repeated measures ANOVA between-group analysis with regard to time was applied; Assumption of normality, homogeneity of variance and compound symmetry were checked and were fulfilled; Post hoc analysis with Bonferroni correction was applied

**Table 3:** Participants’ pain score postextubation on movement between the study groups

Pain score postextubation on movement	Overall^a^	Group		95% CI	*P*-value

		Control^a^	Intervention^a^		
0	6.35 (1.21)	6.95 (0.89)	5.75 (1.21)	6.01, 6.68	< 0.001*
3	4.90 (1.34)	5.85 (0.81)	3.95 (1.05)	4.60, 5.20	< 0.001*
6	4.53 (1.32)	5.40 (0.82)	3.65 (1.14)	4.21, 4.84	< 0.001*
12	4.10 (1.17)	4.90 (0.79)	3.30 (0.92)	3.82, 4.38	< 0.001*
18	3.88 (1.02)	4.45 (0.69)	3.30 (0.98)	3.60, 4.15	< 0.001*
24	3.70 (0.97)	4.45 (0.69)	2.95 (0.51)	3.51, 3.86	< 0.001*
48	3.13 (0.79)	3.60 (0.68)	2.65 (0.59)	2.92, 3.33	< 0.001*

a Mean (SD);

* Statistically significant at *P* < 0.05;

Repeated measures ANOVA between-group analysis with regard to time was applied; Assumption of normality, homogeneity of variance and compound symmetry were checked and were fulfilled; Post hoc analysis with Bonferroni correction was applied

**Table 4:** Participants’ postoperative PCA fentanyl requirement between the study groups

Postextubation PCA fentanyl	Overall^a^	Group		Fentanyl reduction in intervention group	*P*-value

		Control^a^	Intervention^a^		
3 h	215.75 (91.17)	269.50 (81.27)	162.00 (66.14)	24.91 (30.66)	< 0.001*
6 h	194.00 (101.72)	253.00 (103.67)	135.00 (56.24)	30.41 (28.99)	< 0.001*
12 h	172.75 (92.51)	222.00 (90.18)	123.50 (65.80)	28.51 (38.09)	< 0.001*
18 h	150.75 (68.29)	181.50 (80.35)	120.00 (33.56)	20.40 (22.26)	0.003*
24 h	142.50 (84.51)	196.00 (73.58)	89.00 (56.75)	37.54 (39.82)	< 0.001*
48 h	70.25 (86.57)	140.50 (70.67)	0.00 (0.00)	100.00 (00.00)	< 0.001*

a Mean (SD);

* Statistically significantat *P* < 0.05;

PCA = patient controlled analgesia; Repeated measures ANOVA between-group analysis with regard to time was applied; Assumption of normality, homogeneity of variance and compound symmetry were checked and were fulfilled; Post hoc analysis with Bonferroni correction was applied

**Table 5:** Participants’ postoperative spirometry volume between the study groups

Postextubation spirometry volume	Overall^a^	Group		*P*-value

		Control^a^	Intervention^a^	
0 h	25.00 (110.36)	0.00 (0.00)	50.00 (153.90)	0.154
3 h	81.25 (207.14)	0.00 (0.00)	162.50 (272.36)	0.011*
6 h	193.75 (236.75)	50.00 (102.60)	337.50 (247.02)	< 0.001*
12 h	312.50 (281.65)	137.50 (151.20)	487.50 (274.76)	< 0.001*
18 h	387.50 (288.40)	212.50 (146.79)	562.50 (291.04)	< 0.001*
24 h	525.00 (287.56)	337.50 (146.79)	712.50 (272.36)	< 0.001*
48 h	650.00 (264.33)	475.00 (138.13)	825.00 (244.68)	< 0.001*

a Mean (SD);

* Statistically significant *P* < 0.05;

Repeated measures ANOVA between-group analysis with regard to time wasat applied; Assumption of normality, homogeneity of variance and compound symmetry were checked and were fulfilled; Post hoc analysis with Bonferroni correction was applied

**Table 6:** Postoperative patient variables between groups, regardless of time (treatment interaction)

Variables	Control^a^	Intervention^a^	*P*-value
Pain score			
At rest	3.79 (1.14)	2.36 (1.03)	0.031^*^
On movement	5.09 (1.10)	3.65 (1.02)	0.026^*^

PCA fentanyl, mcg/Kg	210.42 (47.69)	104.92 (56.56)	0.006^*^

Spirometry volume, mL	173.21 (180.77)	448.21 (282.51)	0.051

a Mean (SD);

PCA = patient controlled analgesia; Independent *t*-test, *P* < 0.05, indicates a statistically significant difference
